# Staphylococcal Enterotoxin H Induced Apoptosis of Bovine Mammary Epithelial Cells *in Vitro*

**DOI:** 10.3390/toxins6123552

**Published:** 2014-12-19

**Authors:** Yongxia Liu, Wei Chen, Tariq Ali, Rashad Alkasir, Jinhua Yin, Gang Liu, Bo Han

**Affiliations:** Department of Clinical Medicine, College of Veterinary Medicine, China Agricultural University, Yuan Ming Yuan West Road No. 2, Haidian District, Beijing 100193, China; E-Mails: liuyongxia666@163.com (Y.L.); chenwei602@cau.edu.cn (W.C.); tariqkhattak2010@gmail.com (T.A.); rashad.84@hotmail.com (R.A.); yinjinhua@cau.edu.cn (J.Y.); liugang_0402@126.com (G.L.)

**Keywords:** staphylococcal enterotoxin H, expression, bioactivity, bovine mammary gland epithelial cells, viability, apoptosis

## Abstract

Staphylococcal enterotoxins (SEs) are powerful superantigenic toxins produced by *Staphylococcus aureus* (*S. aureus*). They can cause food poisoning and toxic shock. However, their impact on bovine mammary epithelial cells (bMECs) is still unknown. In this study, the distribution of SE genes was evaluated in 116 *S. aureus* isolates from bovine mastitis, and the most prevalent genes were *seh* (36.2%), followed by *sei* (12.1%), *seg* (11.2%), *ser* (4.3%), *sec* (3.4%), *sea* (2.6%) and *sed* (1.7%). To better understand the effect of staphylococcal enterotoxin H (SEH) on bMECs, the *seh* gene was cloned and inserted into the prokaryotic expression vector, pET28a, and transformed into *Escherichia coli* BL21 (DE3). The recombinant staphylococcal enterotoxin H (rSEH) was expressed and purified as soluble protein. Bioactivity analysis showed that rSEH possessed the activity of stimulating lymphocytes proliferation. The XTT assay showed that 100 μg/mL of rSEH produced the cytotoxic effect on bMECs, and fluorescence microscopy and flow cytometry analysis revealed that a certain dose of rSEH is effective at inducing bMECs apoptosis *in vitro*. This indicates that SEs can directly lead to cellular apoptosis of bMECs in bovine mastitis associated with *S. aureus*.

## 1. Introduction

Bovine mastitis is one of the most common economically important diseases in dairy cows throughout the world [1]. Among the etiologic organisms, *Staphylococcus aureus* (*S. aureus*) is recognized as the major pathogen responsible for bovine mastitis, which is contagious in nature [2]. The *S. aureus* can produce a variety of virulence factors, such as surface-associated secretory products, leukotoxins and enterotoxins [3,4]. However, the virulence factors are not distributed uniformly among the *S. aureus* isolates [5].

Staphylococcal enterotoxins (SEs) are low-molecular weight proteins (26.9–29.6 kDa) that are members of the pyrogenic toxin superantigen (PTSAg) family. So far, ten different SEs and twelve staphylococcal enterotoxin-like proteins (SE*l*s) have been described [6,7,8,9,10]. The SEs possess emetic activity and, thus, cause staphylococcal food poisoning and currently include SEA, SEB, SEC, SED, SEE, SEG, SEH, SEI, SER and SET. The SE*l*s toxins, although both homologous and structurally similar to the SEs, either do not induce emesis or have not been confirmed to induce emesis, and these include SElJ, SElK, SElL, SElM, SElN, SElO, SElP, SElQ, SElS, SElU, SElV and SElX [10]. Among these SEs and SE*l*s genes, *sea*, *sec*, *sed*, *see*, *seg*, *seh*, *sei*, *selj*, *selk*, *sell*, *selm*, *seln*, *selo*, *selp*, *selq* and *selu* have been detected in *S. aureus* isolated from raw milk samples in earlier studies [11,12,13].

In the diseases caused by *S. aureus*, cell wall components of *S. aureus* and secreted SEs have been shown to be inflammatory, cytotoxic and septic mediators and can be recognized by the innate immune system via multiple manners [14]. SEs can stimulate non-specific T-cells powerfully and high level cytokine expression by binding to class II major histocompatibility complex (MHC) and T-cell receptor molecules (TCRs) without antigen processing [15,16]. However, the potential role of SEs as virulence factors in bovine mastitis is still unknown [10,17,18,19].

In order to better understand the role of SEs in bovine mastitis, the distribution of SE genes was detected in *S. aureus* isolates from mastitis, and the most prevalent SE gene, *seh*, was cloned and expressed in the prokaryotic expression system. Then, the recombinant protein was purified and prepared and its bioactivity analyzed, and finally, the viability and apoptosis of bovine mammary epithelial cells (bMECs) induced by recombinant staphylococcal enterotoxin H (rSEH) were studied.

## 2. Results and Discussion

### 2.1. Prevalence of SE Genes

*S. aureus* is an important etiologic organism responsible for mastitis in dairy animals. Different *S. aureus* groups are characterized by different virulence factors and by large variations in the presence of these virulence genes [11,13]. In the present study, genes coding the enterotoxins were identified in 116 *S. aureus* isolates associated with bovine mastitis, and the most commonly found was *seh*, which was followed by *sei*, *seg*, *ser*, *sec*, *sea* and *sed* (Table 1). Similar results have been reported from other countries [9,11,20,21], but some differences have been observed in our results. This may indicate that the occurrence of virulence factors can vary among the *S. aureus* strains associated with mastitis in different geographical locations.

**Table 1:** The prevalence of staphylococcal enterotoxins genes in *S. aureus* isolates from bovine mastitis.

Genes	No. of Isolates	% (*n* = 116)
*seh*	42	36.2
*sei*	14	12.1
*seg*	13	11.2
*ser*	5	4.3
*sec*	4	3.4
*sea*	3	2.6
*sed*	2	1.7
*seb*, *see*, *set*	None	0

### 2.2. Sequence Analysis and Expression of the Gene seh

Staphylococcal enterotoxins belong to a large family of pyrogenic exotoxins sharing a common phylogenetic relationship, structure, function and sequence homology. These toxins can cause food poisoning and several allergic and autoimmune diseases [22]. Staphylococcal enterotoxin H was first characterized by Ren *et al.* [23] and, later, was demonstrated to be emetic in monkeys [24]. In the previous studies, the *seh* gene was detected in *S. aureus* isolates from food poisoning [25,26]; even in 1996 and 2005, three outbreaks of food poisoning caused by SEH were reported [27,28,29].

In this paper, among the 42 *seh*-positive *S. aureus* isolates, only two (4.8%) isolates had a single mutation at codon 38 (AGT (Ser) → AGA (Arg)), whereas the rest of the 40 (95.2%) isolates had no mutations when compared with the wild-type (GenBank Accession Number AJ937548.1). DNA sequencing of recombinant plasmids pET28a-SEH also showed that no variation existed in clones of the gene *seh*. All SEs have a similar two-domain topology with a β-barrel motif (up to aa ~126) and a β-grasp motif (from aa ~127) separated by a shallow cavity, and mutations in aa 13–27 affect both MHC class II binding, as well as T-cell interaction [15].

**Figure 1:**
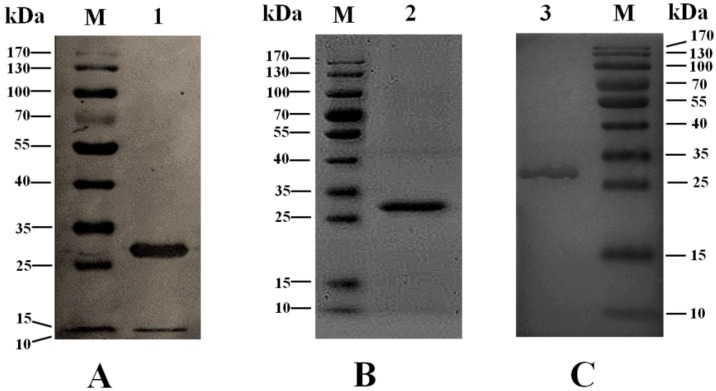
Characterization of recombinant staphylococcal enterotoxin H (rSEH) and the anti-rSEH IgGs. (**A**) Verification of His-tagged rSEH by western blot; (**B**) confirmation of prepared rSEH by 12% SDS-PAGE; (**C**) assessment of the reactivity and specificity of the anti-rSEH IgGs by western blot. Lane M, protein molecular mass marker; Lane 1, His_6_-rSEH fusion protein purified by nickel affinity chromatography; Lane 2, prepared rSEH after digestion and purification; Lane 3, detected rSEH band using the anti-rSEH IgGs.

The gene *seh* was cloned and expressed in the pET28a eukaryotic expression system in our study, the aim of which was to provide an effective tool to study the toxicology of the staphylococcal enterotoxins. For *seh* gene cloning, *Nhe* I and *Xho* І restriction sites were added in the primers to facilitate cloning in the pET28a vector based on the coding sequence of the mature peptide of *seh*, and to improve the ligation efficiency, the target gene was first inserted into the pMD19-T simple vector and verified by restriction digestion and sequencing. The cloned *seh* gene in pET28a is predicted to encode a recombinant protein of 247 aa with a molecular weight of about 27.2 kDa, and the first 23 aa residues at the *N*-terminus (including His-tag) are vector-encoded followed by 224 aa encompassing the target protein. The recombinant fusion protein was expressed with a large quantity under the induction of IPTG and collected as a soluble form. The His-tag was proven to have no effect on the structure of the native protein [30,31,32,33]. The rSEH fusion protein was purified by nickel affinity chromatography and verified by western blot based on His-tag at the N-terminus (Figure 1A). About 2.5 mg of protein were produced from one liter of culture. After digestion, dialysis and chromatography, pure rSEH was prepared in PBS and confirmed by SDS-PAGE (Figure 1B). Through this expression and purification procedure, the rSEH would have six more amino acids (aa) (GSHMAS) at the *N*-terminus compared with the wild-type. Structural analysis by Swiss-Pdb Viewer showed that these six aa have no effect on the structure of SEH, and the polypeptide chain (PPC) composed of these six aa was used as a negative control in the subsequent experiments. Additionally, the anti-rSEH IgGs possessed a high reactivity and specificity of binding with rSEH (Figure 1C). Therefore, the study of the bioactivity and toxicity of staphylococcal enterotoxins can be performed using the recombinant protein. Similar studies have been published using this method [34,35].

### 2.3. Bioactivity of rSEH

**Figure 2:**
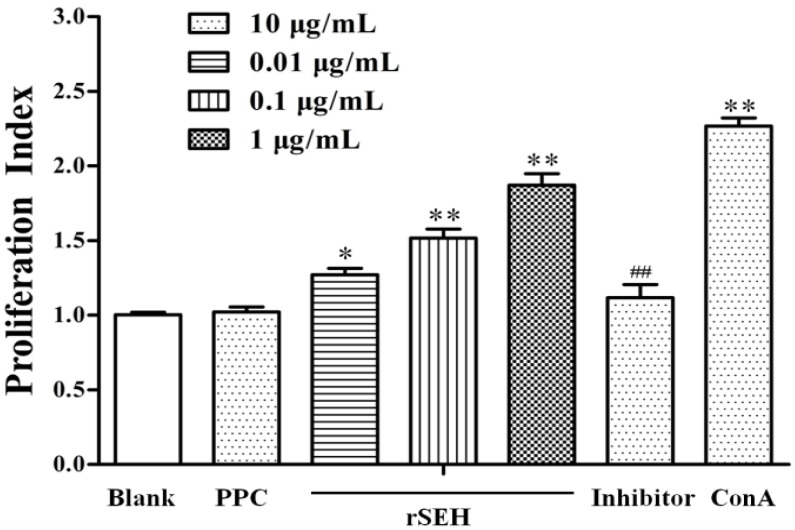
Effect of rSEH on the proliferation of mouse lymphocytes by the XTT assay. The rSEH can promote the proliferation of mouse splenic lymphocytes in a concentration-dependent manner. Blank: blank control; polypeptide chain (PPC): negative control; ConA: positive control; Inhibitor: anti-rSEH IgGs (10 μg/mL) added into the rSEH (1 μg/mL) group. Results are expressed as the mean ± SD (*n =* 6). * *p* < 0.05 and ** *p* < 0.01 as compared with the blank group; ^##^ *p* < 0.01 as compared with the rSEH group.

The results showed that rSEH in different concentrations can promote the proliferation of mouse splenic lymphocytes to different degrees, which was directly correlated with the concentration of rSEH. After exposure to rSEH of 0.01, 0.1 and 1 μg/mL, the cell proliferation index changed to 127.2% ± 4.2% (*p* < 0.05), 151.7% ± 6.1% (*p* < 0.01) and 187.1% ± 7.8% (*p* < 0.01). When we supplemented the anti-rSEH IgGs (10 μg/mL) before exposure to rSEH (1 μg/mL), the cell proliferation index changed to 111.8% ± 8.7% (*p* < 0.01). Furthermore, the six aa at the *N*-terminal of rSEH (102.1% ± 3.5%, *p* > 0.05) were found to have no effect on the proliferation of the cells, and proliferation differences of lymphocytes in rSEH groups were significant or highly significant compared with the negative control (Figure 2).

In a series of previous studies, the staphylococcal enterotoxins were found to share the ability of binding directly to MHC class II molecules on APCs and to TCRs (T-cell receptors) [22]. In the present study, also the SEH was shown to possess this bioactivity; and furthermore, the proliferation of mouse splenic lymphocytes mediated by rSEH was in a dose-dependent manner. Charles Janeway [36] first suggested that the SEs and the determinants encoded by the *Mls* (mouse lymphocyte stimulating) system might act by cross-linking the MHC class II and TCR β-chain, and indeed, SEB was verified by Janice White subsequently [37]. As potent T-cell mitogens, these superantigens possess a wide diversity, exhibiting different preferences for MHC class II alleles and producing distinct TCR Vβ profiles to meet the need of stimulating as many different T-cells as possible while retaining MHC and TCR as target molecules [22]. Since five classical staphylococcal enterotoxins (SEA-SEE) were identified and reported, new variants SEH to SE*l*U have been characterized and designated in the order that they were discovered [22]. The interaction of superantigens with MHC class II can be classified into four groups according to the crystallographic data of their complex: (i) binding to MHC class II α-chain entirely peripheral to the bound antigen peptide (peptide-independent binding), e.g., HLA-DR/SEB; (ii) binding to MHC class II α-chain and extension over the bound peptide (peptide-dependent binding), e.g., HLA-DR/TSST-1; (iii) zinc-mediated binding to MHC class II β-chain and extension over the bound peptide (peptide-dependent binding), e.g., HLA-DR2/SPEC, HLA-DR1/SEH, HLA-DR1/SEI; and (iv) superantigens that combine (i) and (ii) binding modes, such as SEA, to cross-link MHC class II [22].

### 2.4. Effect of rSEH on the Viability of bMECs

**Figure 3:**
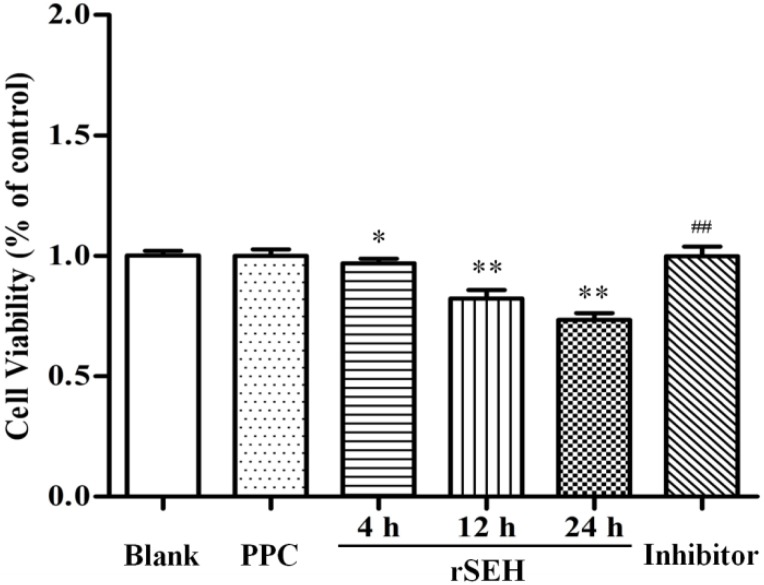
Effect of rSEH on the cell viability of bovine mammary epithelial cells (bMECs) by the XTT assay. The viability of bMECs was significantly decreased compared with the control group when exposed to 100 μg/mL rSEH for 4 h, 12 h and 24 h. Blank: blank control; PPC: negative control; Inhibitor: anti-rSEH IgGs (10 μg/mL) added into the rSEH (24 h) group. Results are expressed as the mean ± SD (*n =* 5). * *p* < 0.05 and ** *p* < 0.01 as compared with the blank group; ^##^ *p* < 0.01 as compared with the rSEH group.

In the results of the XTT assay (Figure 3), the viability of bMECs was significantly decreased as compared with the control group when exposed to 100 μg/mL rSEH for 4 h, 12 h and 24 h, and the cell viability values changed to 96.9% ± 2.0% (*p* < 0.05), 82.2% ± 3.6% (*p* < 0.01) and 73.4% ± 2.9% (*p* < 0.01), respectively. In contrast, pre-treatment with anti-rSEH IgGs (10 μg/mL) in the presence of rSEH elevated cell viability to 99.8% ± 3.1% (*p* < 0.01). This revealed that 100 μg/mL of rSEH possessed a cytotoxic effect on bMECs, and this cytotoxicity developed in a time-dependent manner.

### 2.5. Apoptosis of bMECs Measured by Hoechst-PI Staining Fluorescence Imaging

**Figure 4:**
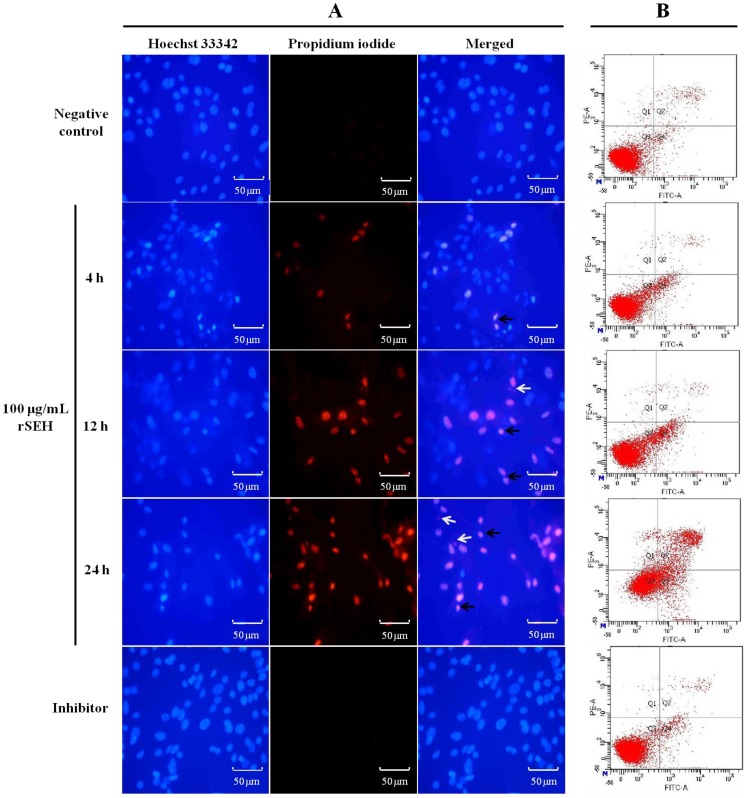
rSEH induces apoptosis in bMECs. (**A**) The bMECs stained with Hoechst 33,342 and PI fluorescent dye after exposure to rSEH (100 μg/mL, 4, 12 and 24 h) and rSEH + anti-rSEH IgGs (10 μg/mL, 24 h). Apoptotic cells were characterized by condensed (black arrow) or fragmented nuclei (white arrow), and the numbers of apoptotic cells increased along with the extension of the incubation time; (**B**) Representative flow cytometric analysis of bMEC apoptotic cells stained for Annexin V and propidium iodide (PI). Negative control: PPC (10 μg/mL); Inhibitor: anti-rSEH IgGs (10 μg/mL) added into the rSEH (24 h) group.

Treated cells were stained with Hoechst 33,342 and PI, and nuclear morphology was evaluated under a fluorescence microscope. As illustrated in the microphotographs (Figure 4A), the nuclei of the dead cells were stained by PI, which released red fluorescence, while the living cells were only stained with Hoechst 33,342 and exhibited blue fluorescence. After exposure to rSEH (100 μg/mL) for 4, 12 and 24 h, the apoptotic cells exhibited condensed and fragmented nuclei, and the amount of apoptotic cells was significantly increased. The cell survival rate changed to 90.5% ± 4.9% (*p* < 0.05), 77.0% ± 4.0% (*p* < 0.01) and 65.5% ± 6.0% (*p* < 0.01) of the negative control. However, these changes were reversed by anti-rSEH IgGs (96.8% ± 6.9%, *p* < 0.01) (Figure 4A).

### 2.6. Apoptosis of bMECs Examined by Flow Cytometry

Flow cytometry assays showed marked changes in cell profiles after exposure to rSEH (100 μg/mL) for 4, 12 and 24 h. This result suggested that rSEH could induce apoptosis in bMECs. All rSEH treatment groups showed significant increases in apoptosis compared with the control group (*p* < 0.01). Apoptotic rates increased from 7.7% ± 1.45% to 36.5% ± 6.37% along with the incubating time of rSEH (Figure 4B). Pre-treatment with anti-rSEH IgGs (10 μg/mL) in the presence of rSEH for 24 h resulted in an observed decrease to 6.3% ± 2.27% (*p* < 0.01).

*S. aureus* is able to internalize into the bovine mammary epithelial cells and can also induce their apoptosis [38]. However, the impact of staphylococcal enterotoxins on bovine mammary epithelial cells is still unknown. The present study is the first report to show the cytotoxicity and pro-apoptotic effect of staphylococcal enterotoxin on bMECs. In the immune response of the mammary gland to *S. aureus*, bMECs are at the front line and play an important role as sentinels; they can respond quickly to bacteria instruction [39]. Some previous studies have shown that several enterotoxins, also defined as cytolysins, can cause cell death by altering the apical membrane permeability of the targeting cells, such as hemolysin, staphylococcal alpha-toxin, pneumolysin and streptolysin-O [40]. They first bind to a host cell membrane and insert a number of water-soluble single-chain polypeptides into the membrane bi-layer, then form the hydrophilic transmembrane pores. These pores would induce necrotic lysis or permeabilization of host cells or intracellular organelles during infection [41]. Among the enterotoxins produced by *S. aureus*, SEB was reported to cause cellular cytotoxicity by inducing cell activation followed by the induction of some specific inflammatory cytokines, chemokines and normal T-cell expressed and secreted proteins [42]; a previous study showed that renal proximal tubule epithelial cells exposed to SEB can be induced to undergo apoptosis [43]. In the same way, SEH was also found to possess the ability of inducing the bMECs to undergo apoptosis, and apoptotic cells increased as time was extended in this study.

## 3. Experimental Section

### 3.1. Detection of SE Genes

One hundred and sixteen *S. aureus* isolates were isolated from cases of bovine mastitis from Beijing and its surrounding area, as described [44]. Bacterial genomic DNA isolation was carried out by the phenol chloroform extraction method [45,46], and their quantity and quality were assessed using the Nanodrop ND-1000 spectrophotometer (Thermoscientific, Wilmington, DE, USA). The presence of SE genes *sea*, *seb*, *sec*, *see*, *sed*, *seg*, *seh*, *sei*, *ser* and *set* was determined by either monoplex or multiplex PCR [8,47,48,49,50]. Amplicons were separated by agarose gel electrophoreses and visualized under an ultraviolet illuminator gel documentation system (Syngene, Frederick, MD, USA) after ethidium bromide (0.5 μg/mL) staining.

### 3.2. Cloning and Expression of the seh Gene

Sequence analysis of the gene *seh* from the *S. aureus* isolates was performed by the procedure given next, and the most predominated *seh* type was used as the donor to amplify the target gene. The mature peptide of the SEH (GenBank Accession Number AJ937548.1) coding region was predicted by Signal P 3.0 Server [51] and amplified with the following primers: forward primer P1: 5'-CGCGCTAGCACATCATATGCGAAAGCAG-3', containing a *Nhe* I site (underlined); and reverse primer P2: 5'-CCGCTCGAGTTATACTTTTTTCTTAGT-3', containing an *Xho* I site (underlined). The PCR protocol used was: initial denaturation at 95 °C for 5 min; 30 cycles of (95 °C, 30 s; 55 °C, 30 s; 72 °C, 1 min); and final extension for 5 min at 72 °C. The amplified PCR product was electrophoresed on 1.2% agarose gel and then gel-purified using an EasyPure^®^ PCR Purification Kit (TransGen Biotech, Beijing, China). Then, the PCR products were inserted into pMD19-T simple vector (Takara, Otsu, Japan). The recombinant plasmid pMD19-SEH was cleavaged with restriction endonucleases *Nhe* I and *Xho* І (Thermo, Waltham, MA, USA), and the DNA fragment coding for mature peptide of SEH was cloned into the *Nhe* I-*Xho* I sites of the pET28a vector (Promega, Madison, WI, USA). The recombinant plasmid pET28a-SEH was transformed into *E. coli* BL21 (DE3)-competent cells (TransGen Biotech, Beijing, China), and the transformants were cultured in Luria-Bertani (LB) nutrient agar (Invitrogen, Carlsbad, CA, USA) containing kanamycin (100 μg/mL). Suspected clones were selected and confirmed by sequencing.

The recombinant protein SEH was expressed in *E. coli* BL21 (DE3) transformed with pET28a-SEH. The transformants were cultured in LB broth (Invitrogen, Carlsbad, CA, USA) containing kanamycin (100 μg/mL) overnight at 37 °C. The pre-culture was used to inoculate fresh LB broth containing kanamycin (100 μg/mL), and the culture was incubated until the OD_600_ reached 0.8. To induce SEH production, isopropyl β-D-thiogalactopyranoside (IPTG) was added to a concentration of 1 mM in the culture broth. The cells were harvested by centrifugation (15 min, 5000× *g*, 4 °C) after induced for 4 h and washed two times with 0.1 M phosphate buffer (pH 7.4, PBS). Then, the cells were lysed in 6× Protein Loading Buffer (TransGen Biotech, Beijing, China) and analyzed by 12% SDS-PAGE [52].

### 3.3. Native Purification and Preparation of rSEH

The bacterial cell pellet was resuspended in lysis buffer (300 mM sodium chloride, 50 mM sodium dihydrogen phosphate, 10 mM imidazole, 10 mM Tris, pH 8.0) and lysed by ultrasonication. Insoluble inclusion bodies and cell debris were removed by centrifugation (30 min, 12,000× *g*, 4 °C). The supernatant was filtered using a 0.45-μm filter (Millipore, Billerica, MA, USA) and loaded onto a 5-mL Ni Sepharose 6 Fast Flow (GE Healthcare, Piscataway, NJ, USA) column; the impurities and unbound proteins were removed by washing the column with lysis buffer. The target proteins were eluted in elution buffer (300 mM sodium chloride, 50 mM sodium dihydrogen phosphate, 300 mM imidazole, 10 mM Tris, pH 8.0), and purification was confirmed by western blot using anti-His monoclonal antibody [53].

The recombinant fusion protein was quantified by the Protein Quantitative Kit (TransGen Biotech, Beijing, China) and digested in elution buffer added with thrombin at 4 °C for 24 h (10 U/mg proteins). The digested sample was dialyzed three times against 0.1 M PBS containing 0.2 M arginine (pH 6.8) at 4 °C for 12 h and applied to a 5-mL Ni Sepharose 6 Fast Flow column to remove the separated His-tag fragment, the uncleaved fusion protein and thrombin. The flow through containing the target protein was collected and desalted with a ZipTip C18 pipette tip (Millipore, Billerica, MA, USA), and the target protein was dissolved in 0.1 M PBS. LPS was detected and removed as previously described [54]. The concentration of rSEH was determined and adjusted to 500 μg/mL. Then, the recombinant protein was confirmed by 12% SDS-PAGE, filtered through a 0.2-μm filter (Millipore, Billerica, MA, USA) and stored at 4 °C prior to use. Anti-rSEH IgGs were prepared as previously described [55], and their reactivity and specificity were detected by western blot.

### 3.4. Bioactivity Analysis of rSEH in Vitro

The ability of purified rSEH to stimulate T-cells was detected as described by Xue *et al.* [56]. ICR (Institute of Cancer Research) mice, outbred strain, weighing 20 ± 1 g, were purchased from Vital River Laboratory Animal Technology Co. Ltd., Beijing, China. The mice were housed and acclimated in a controlled environment (23 ± 2 °C, 50% ± 5% humidity, a natural light-dark cycle) with free access to standard rodent feed (PMI Nutrition International Inc., St. Louis, MO, USA) and tap water before use. A polypeptide chain (PPC) of GSHMAS (*N*'→*C*', l-type) was synthesized from GL Biochem Ltd. (Shanghai, China) and used as the mediator of the negative control. Anti-rSEH IgGs was used to confirm the effect of rSEH on lymphocytes.

Lymphocytes were isolated from ICR mouse spleen and were placed in Roswell Park Memorial Institute 1640 medium (RPMI1640, Hyclone, Logan, UT, USA) with 10% fetal bovine serum (FBS, Hyclone, Logan, UT, USA) and divided into a 96-well plate with a density of 5 × 10^5^ cells per well. The cells were incubated for 48 h at 37 °C with 5% CO_2_, and different stimuli were added based on the grouping method shown in Table 2. After incubation for another 48 hours, the proliferation of the lymphocytes was detected by the XTT Cell Proliferation Assay Kit (Trevigen, Gaithersburg, MD, USA). The values of absorbance were kept in line with the amount of the lymphocytes, and the ability of rSEH to stimulate lymphocyte proliferation was assessed by the following formula:

*x* = (*t* – b)/b (1)

where *x* = proliferation index, *t* = average absorbance of test or control groups and b = average absorbance of blank groups, considering a proliferation index ≥1.5 as the positive group.

**Table 2:** Groups of bioactivity analysis on the ability to stimulate T-cells of rSEH *in vitro.*

Groups	Compositions
Zero adjustment	Culture solution
Blank	Culture solution, splenic lymphocyte
Negative control	Culture solution, splenic lymphocyte, 10 μg/mL PPC
Positive control	Culture solution, splenic lymphocyte, 10 μg/mL ConA
Test	Culture solution, splenic lymphocyte, rSEH at different concentrations (0.01, 0.1, 1 μg/mL)
Inhibition Test	Culture solution, splenic lymphocyte, 1 μg/mL rSEH, and 10 μg/mL anti-rSEH IgGs

### 3.5. Culture and Treatment of Bovine Mammary Epithelial Cells (bMECs)

Primary bovine mammary epithelial cells (bMECs) were isolated and characterized as described previously [57]. Cells were cultured in Cell Culture dishes (Corning, Corning, NY, USA) in growth medium composed of Dulbecco’s modified Eagle’s (DMEM)/F12 medium supplemented with 10% FBS, 100 U/mL penicillin, 100 mg/mL streptomycin and 1 mg/mL amphotericin B. Cells were grown in 5% CO_2_ at 37 °C, and cells from Passages 2–8 were used for these experiments. Cells were cultured to 80% confluence within 2–3 days, and the medium was changed to DMEM/F12 with a low serum content (2% FBS). Then, the cells were exposed to rSEH (100 μg/mL) for 4, 12 and 24 h, respectively. PPC with a final concentration of 100 μg/mL (24 h) was used as the negative control, and anti-rSEH IgGs (10 μg/mL) were supplemented for 1 h as inhibitors before exposure to rSEH in the inhibition assay.

### 3.6. Cell Viability Assay

Cell viability was evaluated with the XTT Cell Proliferation Assay Kit (Trevigen, Gaithersburg, MD, USA). In brief, bMECs were seeded in 96-well plates at a density of 1 × 10^5^ cells per well. After being treated as mentioned above, the cells were washed two times with 0.1 M PBS, and cells in each well were added with 100 μL medium and 25 μL of the activated XTT solution. The plate was maintained at 37 °C for 2 h, and the absorption was measured at 490 nm using a microplate reader (SpectraMax 190, Molecular Devices Corporation, Sunnyvale, CA, USA). Cell viabilities are expressed as the percentages of the control.

### 3.7. Examination of rSEH-Induced Apoptosis by Hoechst-PI Staining Fluorescence Imaging

To assess apoptosis in bMECs, the Hoechst 33342 and propidium iodide (PI) double staining assay was carried out [58]. For this purpose, the Apoptosis and Necrosis Assay Kits (Beyotime Institute of Biotechnology, Nantong, China) were used. Briefly, the treated cells on glass coverslips were washed two times with 2 mL 0.1 M PBS and then incubated with 1 mL of staining buffer containing the Hoechst 33,342 and PI fluorescence dyes in the dark for 30 min at 4 °C. The Apoptosis Inducer Kit (Beyotime Institute of Biotechnology, Nantong, China) was used as the positive control. Afterwards, the stained cells were washed two times and observed under an inverted fluorescence microscope (ECLIPSE Ti-U, Nikon, Tokyo, Japan) at 400× magnification [59]. The percentage of apoptotic cells of the total number was estimated from at least 200 cells randomly counted in five different fields.

### 3.8. Examination of rSEH-Induced Apoptosis by Flow Cytometry

To further corroborate apoptosis induced by rSEH, Annexin V and PI double staining were detected with flow cytometry. The FITC Annexin V Apoptosis Detection Kit I was purchased from Becton Dickinson (Franklin Lakes, NJ, USA). In brief, cells (5 × 10^5^ cells/well) were cultured in 6-well plates. At the end of treatment, the cells were harvested, washed twice with cold PBS and adjusted to 100 μL of 1× Annexin V binding buffer (1 × 10^5^ cells) and transferred to a 5-mL culture tube. Then, 5 μL of Annexin V-FITC and 5 μL of PI were added, and cells were gently vortexed. Cells were then incubated in the dark for 15 min at room temperature (25 °C). Apoptosis rates were determined using a FACSAria flow cytometer (Becton Dickinson) after the addition of 400 μL of 1× binding buffer.

### 3.9. Statistical Analysis

The experiments were repeated three times, and statistical evaluation was carried out using one-way analysis of variance with a statistical software program (Statistical Package for the Social Sciences version 17.0, SPSS, Chicago, IL, USA). Data are presented as the mean values ± standard deviation. *p* ≤ 0.05 was regarded as significant, while *p* ≤ 0.01 was highly significant.

## 4. Conclusions

To the best of our knowledge, this is the first report that staphylococcal enterotoxin H can induce bMEC apoptosis. In Beijing and its surrounding area, the most prevalent SE gene is *seh* in *S. aureus* isolates from mastitis, and the gene *seh* can be expressed as a soluble form in the pET28a prokaryotic expression system. The recombinant SEH possesses the bioactivity of stimulating lymphocyte proliferation, can decrease the viability of bMECs and can induce the cells to undergo apoptosis in a time-dependent manner *in vitro*. This suggests that SEs can directly lead to cellular apoptosis of bMECs in bovine mastitis associated with *S. aureus*.
